# Engineering CXCL10-enriched macrophage-derived extracellular vesicles for T cell recruitment and immune remodeling in pancreatic cancer

**DOI:** 10.1039/d6na00396f

**Published:** 2026-08-04

**Authors:** Shiyao Yin, Ming Li, Yanan Li

**Affiliations:** a College of Pharmaceutical Sciences and Department of Otolaryngology, Children's Hospital, Suzhou Medical College, Soochow University Suzhou 215123 China ynli@suda.edu.cn; b Key Laboratory of Tropical Biological Resources of Ministry of Education, School of Pharmaceutical Sciences, Hainan University Haikou 570228 China mli0919@hainanu.edu.cn; c Song Li's Academician Workstation of Hainan University (School of Pharmaceutical Sciences) Yazhou Bay Sanya 572000 China

## Abstract

CXCL10-enriched macrophage-derived extracellular vesicles were engineered to reprogram the pancreatic tumor microenvironment. CXCL@MEVs enabled bioactive CXCL10 presentation, drove M2-to-M1 macrophage repolarization, recruited and activated CD8^+^ T cells, and suppressed PANC02 tumor growth with minimal systemic toxicity.

## Abstract 2

CXCL@MEVs reprogram TAMs and recruit cytotoxic T cells for PDAC immunotherapy. Image created with BioRender. Yanan, L. (2026). https://BioRender.com/7xmquit.

## Graphical abstract

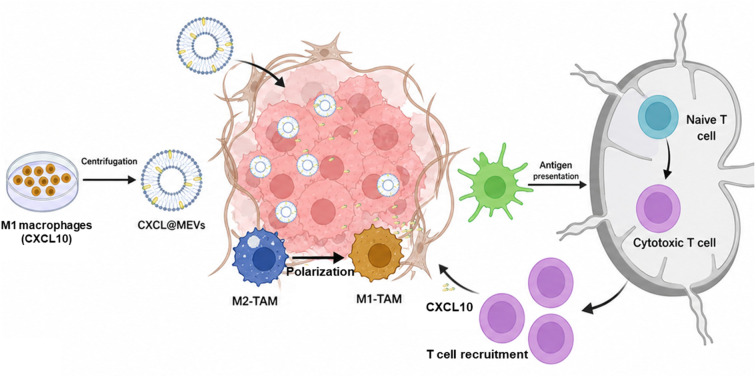

Pancreatic ductal adenocarcinoma (PDAC) remains highly treatment-refractory and is associated with poor clinical outcomes, highlighting the need for more effective therapies.^1–3^ Although immunotherapy has transformed the treatment of several malignancies by engaging innate and adaptive immunity,^4,5^ PDAC responds poorly because its dense stroma and immunosuppressive tumor microenvironment (TME) limit immune-cell access, T-cell infiltration and effector function.^6–8^ In particular, insufficient effector T-cell recruitment and macrophage-dominated immune suppression are major barriers to durable responses. Thus, strategies that remodel the TME while actively recruiting functional effector T cells are important for improving PDAC immunotherapy.

The CXCR3-CXCL9/10/11 axis regulates T-cell trafficking and antitumor immunity.^9–11^ Among these chemokines, CXCL10 is closely associated with T-cell infiltration, improved clinical outcome in melanoma and enhanced T-cell effector activity.^12–16^ This makes CXCL10 an attractive mediator for turning poorly infiltrated tumors into T-cell-enriched lesions. However, direct recombinant CXCL10 administration is limited by instability and rapid clearance, whereas viral-vector-based delivery raises safety concerns and may cause systemic immune stimulation. A delivery strategy that concentrates CXCL10 in tumors while minimizing systemic exposure could therefore improve immune-cell trafficking and activation.

Nanotechnology offers versatile platforms for improving drug delivery and therapeutic specificity.^17–19^ EVs are attractive because their lipid bilayers, endogenous cargo capacity and inherited membrane ligands support biocompatible delivery while retaining parent-cell functions.^19–21^ Macrophage-derived EVs additionally exhibit tumor-homing and immunomodulatory activity.^22–24^ Engineered EVs have been explored for localized delivery of cytokines and immunomodulatory proteins.^25–27^ Here, CXCL10-enriched M1 macrophage-derived EVs were designed as active immunoregulatory carriers that coordinate CXCR3-dependent T-cell recruitment with MEV-driven macrophage remodeling, addressing two complementary immune barriers in PDAC (Scheme 1).

**Scheme 1:**
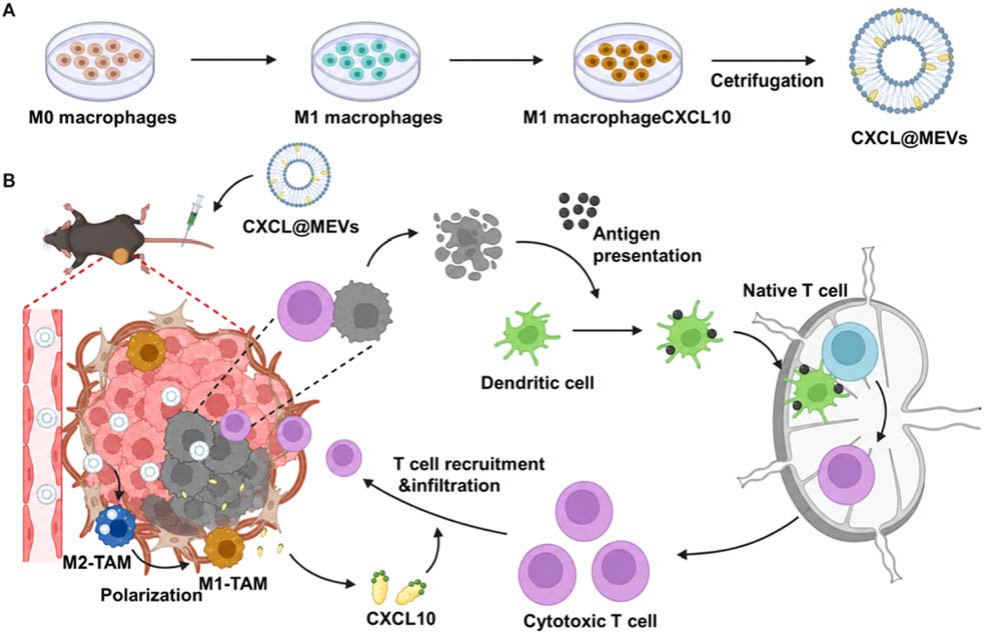
Preparation of CXCL@MEVs (A) and proposed therapeutic mechanism involving intratumoral immune remodeling and T-cell recruitment for pancreatic ductal adenocarcinoma immunotherapy (B). Image created with BioRender. Yanan, L. (2026) https://BioRender.com/uqxeuxl.

The CXCL10 coding sequence (297 bp; NM_021274.2) was cloned into pUC57 and transfected into M1 macrophages to generate CXCL@MEVs (Fig. 1A and B). EGFP fluorescence and CXCL10 quantification confirmed transfection and EV loading (Fig. 1C and D). Purified CXCL@MEVs retained a clear CXCL10 signal after isolation; negligible CXCL10 in the final wash and in pellets obtained from recombinant CXCL10 processed identically excluded appreciable free-protein contamination (Fig. S1, SI). Proteinase K markedly reduced CXCL10 on intact CXCL@MEVs, whereas detergent permeabilization followed by digestion abolished the signal, indicating predominantly surface-accessible CXCL10 with a minor protected fraction (Fig. S2, SI). CXCR3 blockade substantially attenuated CXCL@MEV-induced CD8^+^ T-cell migration, supporting CXCL10-CXCR3-dependent chemotaxis (Fig. S3, SI). TEM showed intact vesicular morphology, and CXCL@MEVs and blank MEVs had similar hydrodynamic diameters (124.1 and 116.1 nm, respectively; Fig. 1E–G). Both vesicle preparations expressed CD9, CD63 and TSG101 but lacked calnexin, supporting EV identity and limited cellular contamination (Fig. S4, SI). CXCL10 content, particle size and zeta potential remained stable after 7 days at 4 °C (Fig. 1H–J), and stored CXCL@MEVs retained chemotactic activity comparable to freshly prepared vesicles (Fig. S5, SI).

**Fig. 1:**
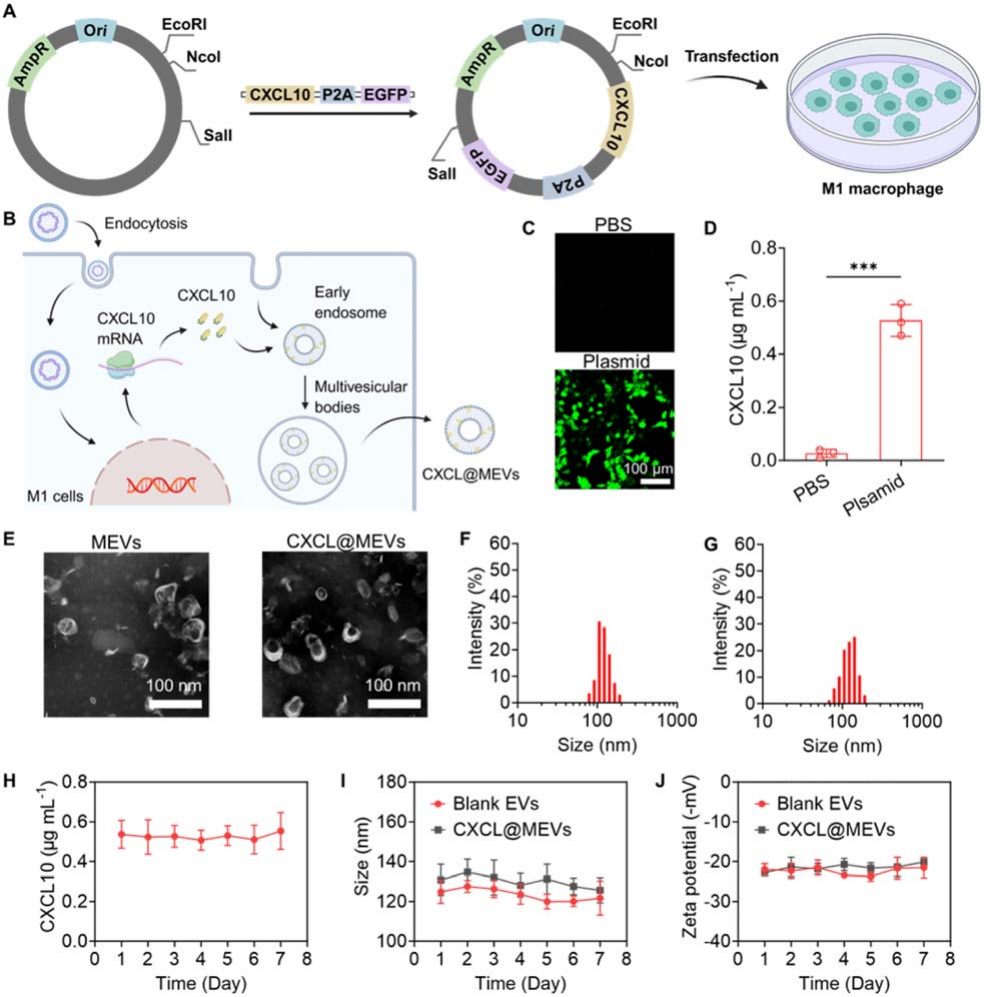
Preparation and characterization of CXCL@MEVs. (A and B) CXCL10 plasmid construction and M1 macrophage transfection. (C) Fluorescence imaging of transfected M1 macrophages. (D) CXCL10 content in CXCL@MEVs and blank MEVs. (E–G) TEM images and particle-size distributions. (H–J) CXCL10 content, particle size and zeta potential of CXCL@MEVs stored at 4 °C for 7 days. Statistical significance was calculated by one-way or two-way ANOVA with Tukey's post hoc test.*P < 0.05, **P < 0.01, ***P < 0.001.

CXCL@MEVs were preferentially internalized by macrophages and dendritic cells (DCs), with limited uptake by PANC02 cells, endothelial cells and fibroblasts (Fig. 2A and B), supporting immune-cell-biased modulation. In M2 macrophages, CXCL@MEVs increased iNOS expression approximately 20-fold *versus* PBS (Fig. 2C and D). Comparable responses to MEVs and CXCL10 + MEVs, but not free CXCL10, indicate MEV-driven repolarization without loss of activity after CXCL10 loading. CXCL@MEV-treated macrophages recruited 4.3 × 10^5^ T cells per well *versus* 4.5 × 10^4^ for PBS, while free CXCL10 induced only modest migration (Fig. 2E and F). They also increased T-cell IFN-γ by 11.4-, 5.3- and 4.7-fold relative to PBS, CXCL10 and CXCL10 + MEVs, respectively (Fig. 2G), and reduced PANC02 tumor-cell viability to 27.7% (Fig. 2H). Conditioned medium from this co-culture increased DC CD80/CD86 expression (Fig. 2I, J and S8, SI). Together, these data support coordinated MEV-mediated macrophage remodeling and CXCL10-associated T-cell recruitment, although the specific EV surface ligands or luminal cargo molecules responsible for macrophage repolarization remain to be further identified.

**Fig. 2:**
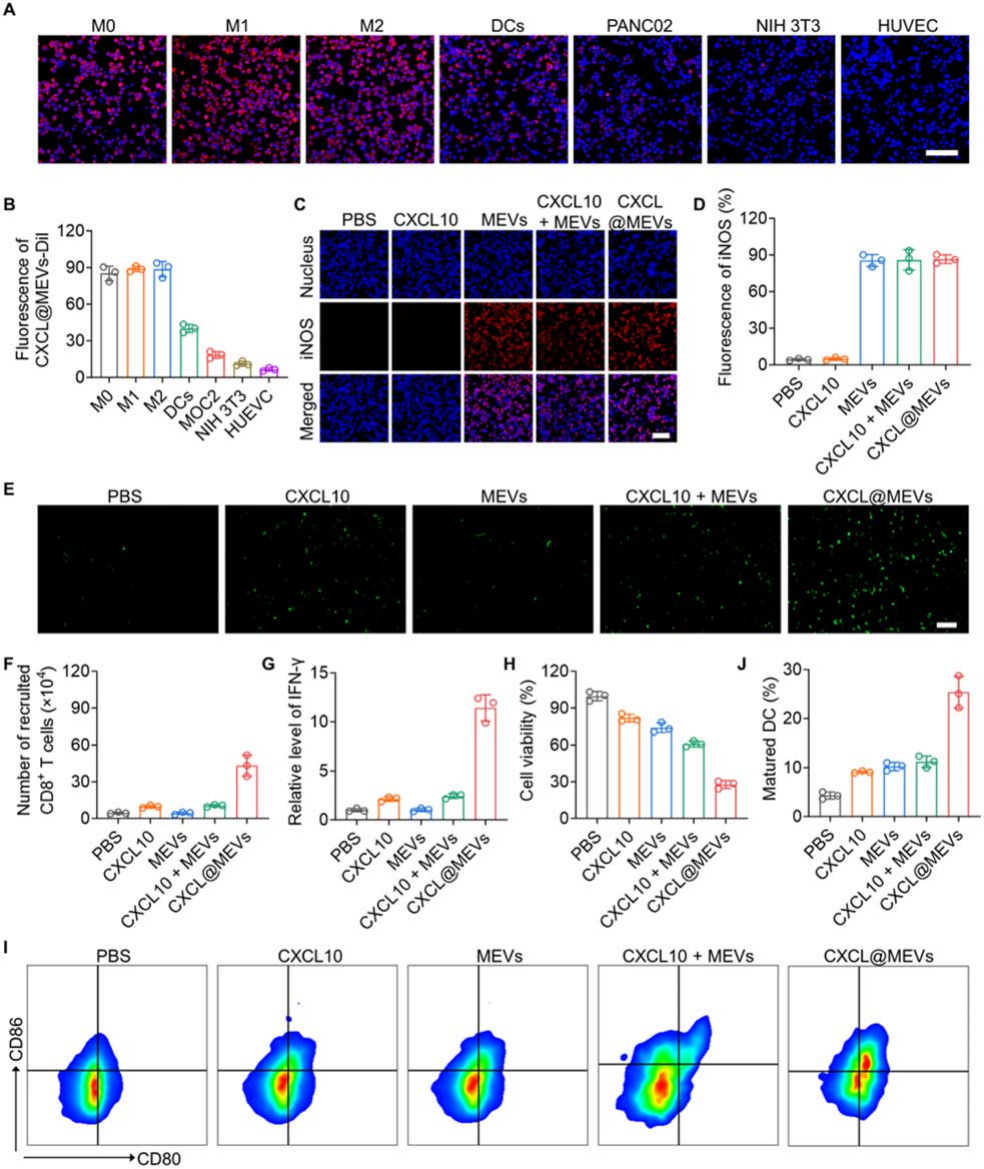
*In vitro* cellular uptake, macrophage polarization, T-cell recruitment and DC maturation. (A and B) Fluorescence images and quantification of CXCL@MEV uptake by different cells. (C and D) iNOS expression in M2 macrophages after the indicated treatments. (E and F) Representative images and quantification of recruited T cells. (G) IFN-γ levels of T cells co-cultured with pretreated M2 macrophages. (H) PANC02 tumor-cell viability after co-culture with pretreated M2 macrophages. (I and J) CD80/CD86 expression in DCs treated with conditioned medium. Scale bar, 100 µm. Statistical significance was calculated by one-way ANOVA with Tukey's post hoc test.

At 24 h after intravenous administration, DiR-labelled CXCL@MEVs showed greater tumor-associated fluorescence than free DiR. However, substantial hepatic uptake was also observed, with a tumor-to-liver fluorescence ratio of 0.93. Liver fluorescence was comparable to tumor fluorescence (*P* = 0.661), indicating appreciable tumor-associated accumulation, although without preferential distribution over the liver (Fig. S6, SI). In the same PANC02 model, flow cytometry demonstrated that CXCL@MEVs promoted TAM repolarization. The proportion of CD206^+^ M2-like TAMs was approximately 2.6-fold lower than that in the PBS group, whereas CXCL10, MEVs, and CXCL10 + MEVs produced only modest reductions. Conversely, CD86^+^ M1-like TAMs increased by 5.3-, 4.1-, 2.7-, and 2.3-fold relative to PBS, CXCL10, MEVs, and CXCL10 + MEVs, respectively (Fig. 3A–D). These results indicate that CXCL@MEVs retain the macrophage-reprogramming capability of MEVs *in vivo* and induce robust M2-to-M1-like TAM repolarization. CXCL@MEVs also increased CD3^+^CD8^+^ T cells to 17.2% of tumor immune cells, representing 2.2- and 4.7-fold increases over CXCL10 + MEVs and PBS, and elevated intratumoral IFN-γ^+^ T cells (Fig. 3E–H). Immunofluorescence confirmed enhanced CD8^+^ T-cell infiltration, consistent with the establishment of a CXCL10-associated recruitment signal within tumors (Fig. 3I). In tumor-draining lymph nodes, CD11c^+^CD80^+^CD86^+^ DCs increased to 21.1%, exceeding CXCL10 (6.5%), MEVs (8.2%), CXCL10 + MEVs (11.0%) and PBS (3.8%) (Fig. 3J), indicating strengthened antigen-presenting activity. CXCL@MEVs further reduced Tregs and MDSCs by 3.1- and 2.9-fold *versus* PBS (Fig. S9, SI), supporting coordinated immune activation and relief of immunosuppression.

**Fig. 3:**
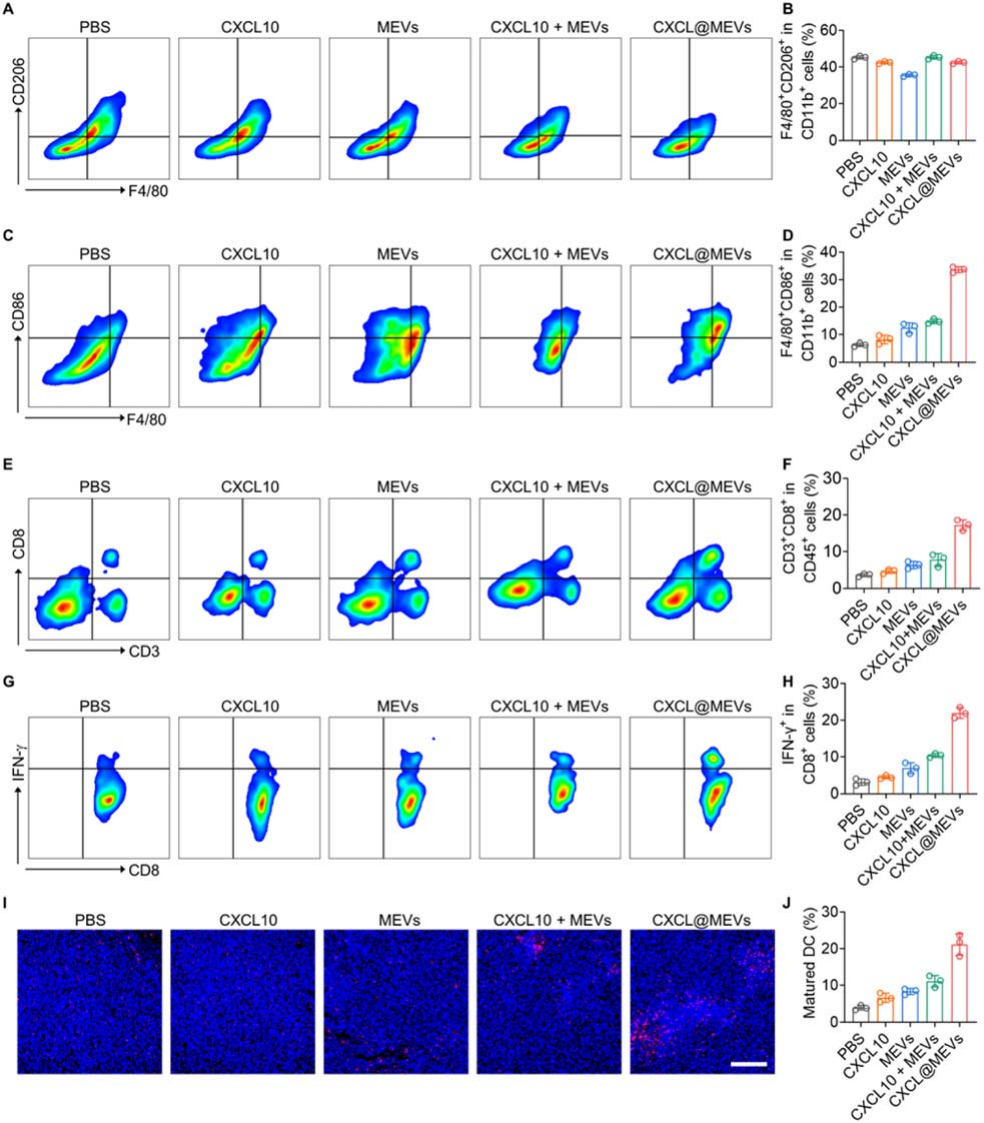
*In vivo* immune activation by CXCL@MEVs. Representative flow cytometry plots and quantification of F4/80^+^CD206^+^ macrophages (A and B), F4/80^+^CD86^+^ macrophages (C and D), CD3^+^CD8^+^ cytotoxic T cells (E and F) and IFN-γ^+^ T cells (G and H) in PANC02 tumors after the indicated treatments. (I) Representative fluorescence images of T-cell infiltration in tumor tissues. (J) Quantification of DC maturation in tumor-draining lymph nodes. Scale bar, 100 µm. Statistical significance was calculated by one-way or two-way ANOVA with Tukey's post hoc test.

Therapeutic efficacy was evaluated in subcutaneous PANC02 tumor-bearing mice treated intravenously on days 0, 3 and 6 after tumors reached approximately 100 mm^3^ (Fig. 4A). CXCL10, MEVs and CXCL10 + MEVs showed modest tumor inhibition (10.5%, 18.5% and 32.5% on day 21), whereas CXCL@MEVs achieved 69.1% inhibition and reduced average tumor volume 2.6-fold compared with CXCL10 + MEVs by day 24 (Fig. 4B and C). The superior efficacy of CXCL@MEVs over the physical mixture supports the importance of co-packaging for coordinated delivery and immune modulation. CXCL@MEVs also extended median survival to 36 days (Fig. 4D), with no evident body-weight loss (Fig. S10, SI), indicating improved therapeutic efficacy and preliminary tolerability. H&E staining showed extensive tumor necrosis and inflammatory changes after CXCL@MEVs treatment, and Ki67/TUNEL staining further confirmed stronger tumor damage, with a 4.1-fold decrease in Ki67^+^ cells and a 7.7-fold increase in TUNEL^+^ cells compared with controls (Fig. 4E–G and S11, SI). CXCL@MEV treatment resulted in the highest total intratumoral CXCL10 level, (Fig. 4H), which may reflect a combination of EV-associated CXCL10 delivery and treatment-induced endogenous CXCL10 production. Tumor TNF-α and IFN-γ increased by 2.9- and 4.0-fold over PBS, respectively (Fig. 4I and J), further supporting CXCL@MEVs-driven immune activation.

**Fig. 4:**
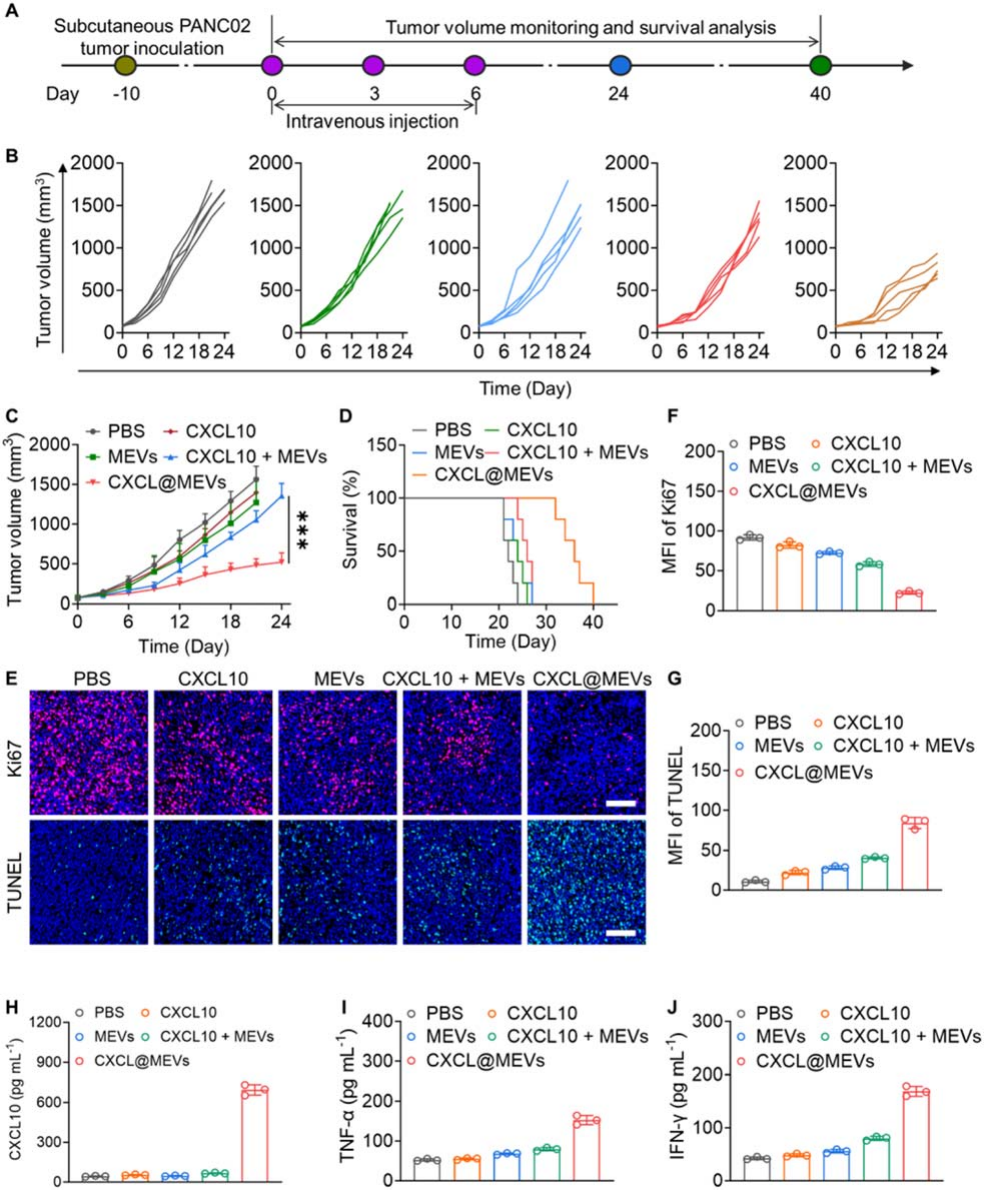
*In vivo* antitumor effects of CXCL@MEVs. (A) Treatment timeline. (B and C) Individual and average tumor growth curves of PANC02 tumor-bearing mice. (D) Kaplan–Meier survival curves. (E–G) TUNEL and Ki67 staining and quantification in tumor tissues. (H–J) CXCL10, TNF-α and IFN-γ levels in tumors. Scale bar, 100 µm. Statistical significance was calculated by one-way or two-way ANOVA with Tukey's post hoc test. *P < 0.05, **P < 0.01, ***P < 0.001.

CXCL@MEVs caused minimal hemolysis (2.4%) and did not alter liver/renal function, blood counts or major-organ histology *versus* PBS, supporting preliminary biosafety under the tested conditions (Fig. S12–S17, SI).

In summary, component-control experiments indicated separable but complementary functions: M1 macrophage-derived EVs primarily promoted M2-to-M1-like macrophage repolarization, whereas CXCL10 mediated CXCR3-dependent T-cell chemotaxis. Previous studies have similarly shown that M1 macrophage-derived vesicles can activate pro-inflammatory signaling and reprogram M2-like macrophages, supporting their function as active immunoregulatory carriers rather than passive vehicles.^28^ Surface-associated CXCL10 on EVs has also been reported to engage CXCR3,^29^ consistent with our proteinase K accessibility and CXCR3-blocking results. However, because the current stability analysis was limited to storage at 4 °C, the stability of surface-accessible CXCL10 in serum or during systemic circulation remains unknown. The stronger T-cell chemotactic activity and antitumor efficacy of CXCL@MEVs relative to the physical mixture support the functional value of CXCL10 association with MEVs, but do not establish molecular synergy. The specific MEV cargo responsible for macrophage remodeling and the effects of CXCL10 association on EV composition or CXCR3 signaling remain to be determined.

Unlike EV platforms that directly remodel the PDAC stroma,^30^ CXCL@MEVs were not shown to modify the fibrotic compartment. Although they increased CD8^+^ T-cell infiltration in the subcutaneous PANC02 model, their ability to function within stromal-rich orthotopic tumors remains uncertain. The 24 h biodistribution data showed tumor-associated fluorescence together with substantial hepatic uptake; however, this single time point does not define the pharmacokinetic or clearance profile of CXCL@MEVs. Overall, CXCL@MEVs enhanced TAM repolarization, CD8^+^ T-cell infiltration, DC maturation, and tumor suppression, supporting a proof-of-concept active-carrier strategy for coordinating complementary immune responses. Further studies should identify the active MEV cargo, distinguish delivered from endogenously induced CXCL10, and validate efficacy in orthotopic PDAC models.

## Author contributions

Shiyao Yin: investigation, validation, formal analysis, software, data curation, writing – original draft. Ming Li: methodology, visualization, resources, funding acquisition, supervision, writing – review and editing. Yanan Li: conceptualization, supervision, project administration, writing – review and editing.

## Conflicts of interest

There are no conflicts to declare.

## Supplementary Material

NA-OLF-D6NA00396F-s001

## Data Availability

The data supporting this article have been included as part of the supplementary information (SI). Supplementary information is available. See DOI: https://doi.org/10.1039/d6na00396f.
