# Supplementary material for: Engineering CXCL10-enriched macrophage-derived extracellular vesicles for T cell recruitment and immune remodeling in pancreatic cancer
Source: Nanoscale Adv. 2026 Aug 4;8(17):4776–80. doi: 10.1039/d6na00396f (PMC13464418; doi:10.1039/d6na00396f)
Supplement: Supplementary file 1 [file NA-008-D6NA00396F-s001.pdf]

Supplementary Information (SI) for Nanoscale Advances.
This journal is © The Royal Society of Chemistry 2026

Supporting Information

Engineering CXCL10-Enriched Macrophage-Derived Extracellular Vesicles for T Cell Recruitment and Immune Remodeling in Pancreatic Cancer

Shiyao Yin,[a] Ming Li*[abc] and Yanan Li*[a]

[a]College of Pharmaceutical Sciences and Department of otolaryngology, Children's Hospital, Suzhou Medical College, Soochow University, Suzhou 215123, China

[b] Key Laboratory of Tropical Biological Resources of Ministry of Education, School of Pharmaceutical Sciences, Hainan University, Haikou 570228, China

[c]Song Li's Academician Workstation of Hainan University (School of Pharmaceutical Sciences), Yazhou Bay, Sanya 572000, China

Contents

1. Experimental Materials and Methods

2. Supporting Figures

1. Experimental Materials and Methods

Materials and cells.

Hoechst 33342 was obtained from Life Technologies (California, USA). Dulbecco's Modified Eagle Medium (DMEM) and RPMI 1640 were acquired from Cytiva Life Sciences (Hyclone, Utah, USA). Protease and phosphatase inhibitor cocktails were obtained from TargetMol Chemicals Inc. (Boston, MA, USA). Sinopharm Chemical Reagent Co., Ltd. (Shanghai, China) supplied additional reagents and materials. PANC02 cells, DC2.4 mouse dendritic cells, and RAW 264.7 mouse macrophages were obtained from IMMOCELL (Xiamen, Fujian, China). DC2.4 cells were cultured in RPMI 1640, while other cells were maintained in DMEM, supplemented with 10% fetal bovine serum (FBS), and incubated at 37 °C and in a 5% CO2 atmosphere.

Animal handling.

Animal studies were conducted using female C57BL/6J mice (6 - 8 weeks old, 20 ± 2 g body weight; SLAC Laboratory Animal Co., Shanghai) housed under controlled conditions (21 ± 1 °C, 40 - 70% humidity) with 12-hour light/dark cycles. The study followed approved animal care protocols as authorized by the Institutional Animal Care and Use Committee (approval no. ECSU-202408A0068). All experimental procedures were conducted in compliance with established ethical guidelines for animal research. After tumor volumes reached 70–100 mm^3, mice were randomly assigned to different treatment groups with comparable baseline tumor volumes. Tumor volume and body weight were measured according to the same predefined procedure throughout the study. For flow-cytometry, immunohistochemical, immunofluorescence and histological analyses, tumor and organ samples were coded before processing and quantification where applicable.

Synthesis and characterization of CXCL@MEVs.

Plasmid construction. The CXCL10 coding sequence (297 bp; NM_021274.2) was PCR-amplified using NcoI/SalI-tailed primers and cloned into the pUC57-EGFP vector. Following restriction digestion (NcoI/SalI), the fragment was directionally ligated into the corresponding sites upstream of the EGFP reporter. Recombinant plasmids were transformed into DH5α competent cells (E. coli), with positive clones verified through colony PCR, restriction mapping, and Sanger sequencing to confirm proper insertion and reading frame preservation.

Extracellular vesicles (EVs) production and isolation. The plasmid containing the CXCL10 sequence was transfected into M1 macrophages using Lipofectamine 2000. After a 24-hour transfection period, the cells expressing CXCL10 were subjected to ultraviolet (UV) irradiation for 1 hour and then incubated with fresh culture medium for 12 hours. EV-enriched supernatants were sequentially centrifuged to remove cellular debris (400 × g, 10 min), large apoptotic bodies (2,000 × g, 15 min), and microvesicles (10,000 × g, 30 min). CXCL@MEVs were purified by ultracentrifugation (100,000 × g, 2 h, Optima L-90K ultracentrifuge, Beckman Coulter). Parallel preparations yielded control naive MEVs (untreated), which were subjected to identical isolation protocols.

Transmission electron microscopy (TEM, Tecnai G20, FEI) revealed spherical morphology of all EV variants (blank MEVs, CXCL@MEVs). Dynamic light scattering (Zetasizer ZS90, Malvern Instruments) quantified hydrodynamic diameter and zeta potential at 25 °C.

EV dosing and normalization.

EV protein concentration was determined using a BCA protein assay and was used for dose normalization in all in vitro and in vivo experiments. Particle size and zeta potential were used for EV characterization, whereas particle number was not used as the dosing parameter. Unless otherwise stated, MEVs, CXCL@MEVs and the MEV component in the CXCL10 + MEVs physical mixture were administered at equivalent EV protein doses. For in vitro experiments, EVs were used at 200 μg mL^{-1} EV protein. For in vivo experiments, mice were intravenously administered EVs at 5 mg kg^{-1} EV protein. The CXCL10 loading amount in CXCL@MEVs was 126.4 ± 18.7 pg CXCL10 per μg EV protein. Therefore, the recombinant CXCL10 dose used in the free CXCL10 and CXCL10 + MEVs groups was matched to the CXCL10 content in the corresponding CXCL@MEV dose.

Cellular uptake

M0, M1, and M2 macrophages, DCs, PANC02 cells, NIH 3T3 cells, and HUVECs were seeded on the confocal dishes (5.0×10^5 cells/well) and incubated overnight in a culture medium containing 10% FBS. DiI-labeled CXCL@MEVs (200 μg mL^{-1} EV protein) were added to the wells. After 12 h incubation, the cells were washed three times using PBS. Then, the cells were incubated with 0.5 mL of Hoechst 33342 (10 μg mL^{-1}) for 15 min. Then, the cells were fixed, and the confocal imaging was performed using a confocal laser scanning microscope (CLSM, Zeiss LSM710, Oberkochen, Germany).

CXCL10 quantification.

CXCL10 levels in CXCL@MEVs and tumor tissues were determined using a mouse CXCL10/IP-10 ELISA kit (R&D Systems, Cat. No. MCX100) according to the manufacturer's instructions. Briefly, purified EVs were washed with PBS, lysed on ice using a mild detergent-containing lysis buffer supplemented with protease inhibitors, and centrifuged at 12,000 × g for 10 min at 4 °C. Tumor tissues were weighed, homogenized in ice-cold lysis buffer containing protease inhibitors, and centrifuged at 12,000 × g for 15 min at 4 °C. The resulting supernatants were appropriately diluted and subjected to ELISA analysis. Absorbance was measured at 450 nm with wavelength correction at 540 or 570 nm. CXCL10 concentrations were calculated from a recombinant mouse CXCL10 standard curve and normalized to EV protein content or tumor tissue protein content.

Proteinase K protection assay

To determine the topology of CXCL10 associated with CXCL@MEVs, purified CXCL@MEVs were divided into equal aliquots and treated with PBS, Triton X-100, proteinase K, or Triton X-100 plus proteinase K. Proteinase K treatment was used to digest surface-accessible proteins, whereas Triton X-100 was used to

permeabilize the vesicle membrane and expose protected intravesicular proteins. After digestion, proteinase K activity was terminated by adding protease inhibitor. CXCL10 levels in each group were quantified using a CXCL10 ELISA kit according to the manufacturer's instructions.

Macrophage polarization.

M2 macrophages were seeded on the confocal dishes (5.0×10^5 cells/well) and incubated overnight in a culture medium containing 10% FBS. PBS, MEVs, CXCL10, CXCL10 + MEVs, and CXCL@MEVs were added to the dishes and incubated for 12 h (EVs were used at 200 μg mL^{-1} EV protein). Then, the cells were washed with PBS and fixed with 4% paraformaldehyde (PFA), followed by immunofluorescence staining of iNOS using anti-iNOS antibody and Cy5-labeled second antibody. After that, the cells were washed, and the confocal imaging was performed using a confocal laser scanning microscope (CLSM, Zeiss LSM710, Oberkochen, Germany).

T cell isolation and recruitment.

C57BL/6J mice were euthanized by cervical dislocation, with spleens aseptically harvested following 70% ethanol disinfection (15 min). Splenocytes were obtained by mechanical dissociation (70 μm nylon mesh filtration, Biosharp) and erythrocyte lysis (Beyotime). $CD3^+$ T cells were magnetically isolated and cultured in complete RPMI 1640 (10% FBS, 1 mM sodium pyruvate, 10 mM HEPES, 55 μM β-mercaptoethanol) with 10 ng mL-1 IL-2 (PeproTech). For activation, cells were stimulated with plate-bound anti-CD3/CD28 antibodies (5 μg mL-1 each, BioLegend) for 48 h.

In 24-well Transwell systems (5 μm pores, Corning), 2×10^6 activated T cells were seeded in serum-free medium (upper chamber), while M2 macrophages pretreated with various treatments (PBS, MEVs, CXCL10, CXCL10 + MEVs, and CXCL@MEVs) were placed in the lower chamber. After 12 h of migration, transmigrated cells were fixed (4% PFA, 15 min), stained with anti-CD3 and anti-CD8 antibodies, and analyzed by flow cytometry using a BD system (ARAIII).

CXCR3-blocking Transwell migration assay

To evaluate the contribution of CXCR3 to CXCL@MEV-mediated migration, $CD8^+$ T cells were pretreated with an anti-CXCR3 blocking antibody at 37 °C for 30 min before being seeded into the upper chambers of Transwell inserts. PBS, recombinant CXCL10, MEVs or CXCL@MEVs were added to the lower chambers. After incubation at 37 °C for 3-4 h, migrated $CD8^+$ T cells in the lower chambers were collected and quantified by flow cytometry.

Flow cytometry analysis.

$CD3^+$ T cells and M2 macrophages pretreated with various treatments (PBS, MEVs, CXCL10, CXCL10 + MEVs, and CXCL@MEVs) were co-cultured at a T cell/M2 macrophage ratio of 10:1. After 5 days of co-culture, cells were collected, fixed, permeabilized, and stained with fluorescence-conjugated anti-IFN-γ antibody (1:100 dilution; BioLegend) for intracellular cytokine analysis. Samples were washed, resuspended in 500 µL of ice-cold FACS buffer (PBS containing 1% BSA and 0.1% sodium azide), and analyzed by flow cytometry using a BD system (ARAIII).

Cytotoxicity.

M2 macrophages pretreated with various treatments (PBS, MEVs, CXCL10, CXCL10 + MEVs, and CXCL@MEVs) were co-cultured with PANC02 cells at a tumor cell/M2 macrophage ratio of 10:1. Following 5 days of co-culture, cells were digested, collected, and labeled with anti-CD45, and used magnetic beads to separate macrophages from cancer cells. Tumor cell viability was assessed using a CCK-8 assay, with the PBS group used as the control for calculating relative cell survival in each group.

***In vitro* BMDC maturation.**

M2 macrophages pretreated with various treatments (PBS, MEVs, CXCL10, CXCL10 + MEVs, and CXCL@MEVs) were co-cultured with PANC02 cells at a tumor cell/M2 macrophage ratio of 10:1. Following 5 days of co-culture, the supernatants were collected and added to the immature BMDCs. After 24 h, BMDCs were stained with anti-CD11c, anti-CD80, and anti-CD86 antibodies. Then, samples were vortexed, centrifuged at 500 × g for 3 minutes, and the supernatant was carefully aspirated before resuspension in 500 µL of ice-cold FACS buffer (PBS containing 1% BSA and 0.1% sodium azide) for immediate flow cytometric analysis using a BD system (ARAIII).

Establishment of subcutaneous PANC02 tumor model.

Female C57BL/6J mice (6 - 8 weeks old, 18 - 20 g, SLAC Laboratory Animal Co., Ltd., Shanghai) were acclimatized for one week before experimentation. Tumorigenesis was induced by subcutaneous injection of 5×10^6 PANC02 cells (50 µL PBS suspension) into the right dorsal flank using a syringe. Tumor growth was monitored by digital caliper measurements, with volume calculated as (length × $width^2$)/2. Treatment protocols were initiated when tumors reached 70 - 100 mm^3 (typically 10 days post-inoculation), with randomization ensuring equivalent baseline tumor volumes across experimental groups.

***In vivo* antitumor immune response.**

PANC02 tumor-bearing C57BL/6J mice (tumor volume 70 - 100 mm^3) were randomized into 5 treatment groups receiving intravenous injections of PBS (vehicle control), MEVs (empty vector control), CXCL10, CXCL10 + MEVs, and CXCL@MEVs (EVs were administered at 5 mg kg^{-1} EV protein, n = 3 per group). At 72 hours post-treatment, tumors and draining lymph nodes were aseptically excised and enzymatically digested (Collagenase IV, 1 mg/mL; DNase I, 100 U/mL, 37 °C, 60 min) to generate single-cell suspensions. Immune cell phenotyping was conducted using flow cytometry. Lymph node-derived cells were stained with anti-CD11c, anti-CD80, and anti-CD86 antibodies for 2 hours to assess dendritic cell maturation. Tumor-derived cells were stained for 2 hours with anti-CD45, anti-CD11b, anti-F4/80, anti-CD206, anti-CD80, and anti-Gr-1 antibodies to evaluate the presence of M2-like, M1-like tumor-associated macrophages (M2/M1 TAMs) and myeloid-derived suppressor cells (MDSCs). Additionally, cells from tumors were also stained with anti-CD45, anti-CD3, and anti-CD8 to analyze cytotoxic T lymphocytes (CTLs), respectively. Samples were then vortexed, centrifuged at 500 × g for 3 minutes, and the supernatant was carefully aspirated before resuspension in 500 µL of ice-cold FACS buffer (PBS containing 1% BSA and 0.1% sodium azide) for immediate flow cytometric analysis using a BD system (ARAIII).

***In vivo* antitumor efficacy.**

To assess the therapeutic efficacy of CXCL@MEVs, C57BL/6 mice bearing established subcutaneous PANC02 tumors (70-100 mm^3 initial volume) were randomized into different treatment groups (n = 5 per group) receiving intravenous injections of PBS (vehicle control), MEVs (empty vector control), CXCL10, CXCL10 + MEVs, and CXCL@MEVs on days 0, 3, and 6. Tumor dimensions were measured every 3 days using digital calipers, with volumes calculated as $V = (L \times W^2)/2$, where L represents the longest diameter and W the perpendicular shortest diameter. Animals were humanely euthanized by CO2 asphyxiation followed by cervical dislocation when tumors reached the ethical endpoint volume of 1500 mm^3 or if ulceration occurred, with the study duration extending to 40 days post-treatment initiation to evaluate long-term therapeutic effects.

Serum biochemistry assay.

Healthy C57BL/6J mice (n = 3 per timepoint) received intravenous administration of CXCL@MEVs at the experimental dose used for the corresponding in vivo studies, with blood samples collected via retro-orbital bleeding at predetermined intervals (1-, 3-, 7-, and 14-days post-injection) for comprehensive haematological and biochemical analysis. Complete blood counts were performed using an automated haematology analyser (Sysmex XN-1000) to quantify leukocyte, erythrocyte, and platelet parameters. Serum biochemistry profiles were obtained by centrifugation (3000 × g, 10 min, 4 °C) followed by analysis of hepatic function markers (aspartate aminotransferase, AST; alanine aminotransferase, ALT; alkaline

phosphatase, ALP) and renal function indicators (blood urea nitrogen) using a clinical chemistry analyser (Hitachi 7180) according to standardized manufacturer protocols.

Statistical analysis.

All quantitative data are expressed as mean ± standard deviation (SD) for technical replicates or mean ± standard error of the mean (SEM) for biological replicates, as appropriate. Statistical analyses were performed using GraphPad Prism software (version 9.0; GraphPad Software, San Diego, CA), with group comparisons assessed by either one-way ANOVA (for single-factor experiments) or two-way ANOVA (for multifactorial designs), followed by Tukey's honest significant difference post hoc test for multiple comparisons. The threshold for statistical significance was defined as $^{*}P < 0.05$, $^{**}P < 0.01$, and $^{***}P < 0.001$.

2. Supporting Figures

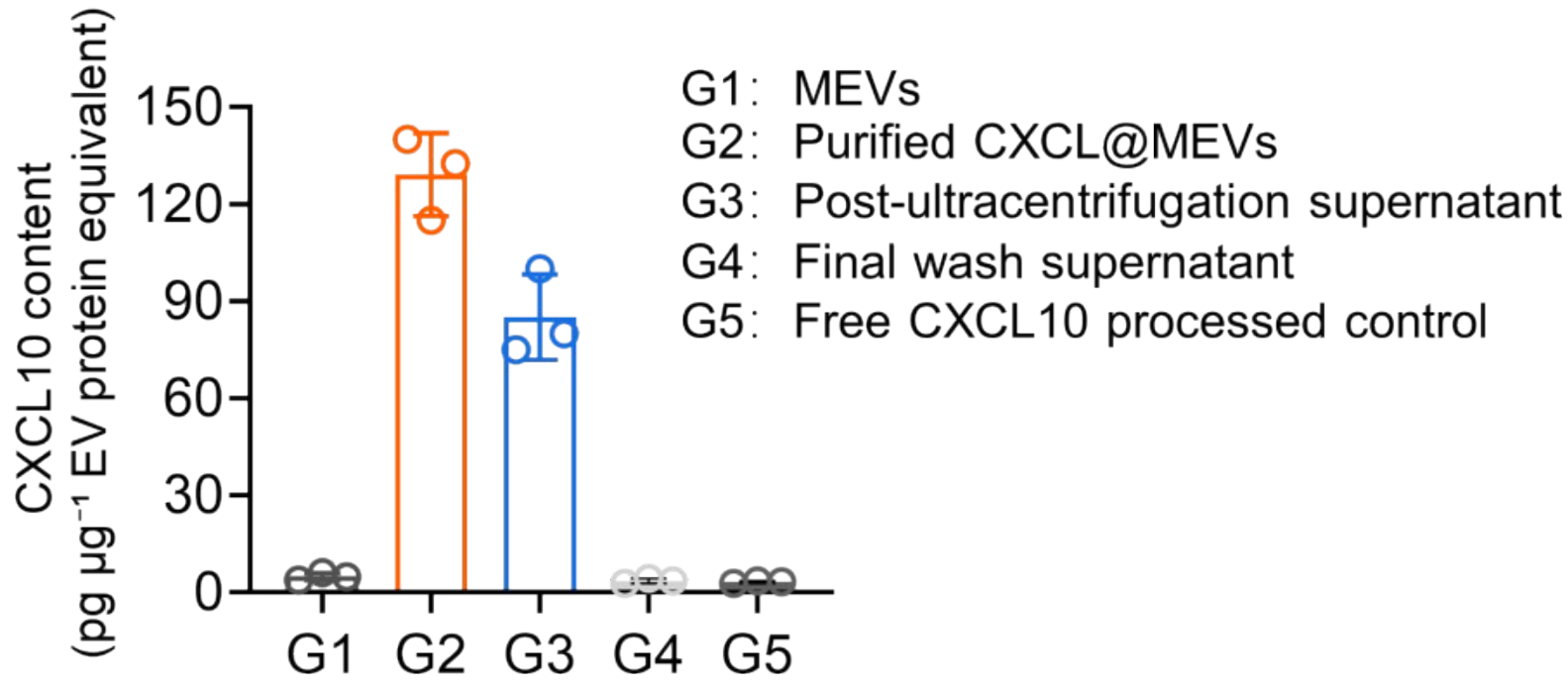

Fig. S1. CXCL10 levels in different fractions during ultracentrifugation-based purification were quantified by ELISA. Data are shown as mean ± SD, n = 3.

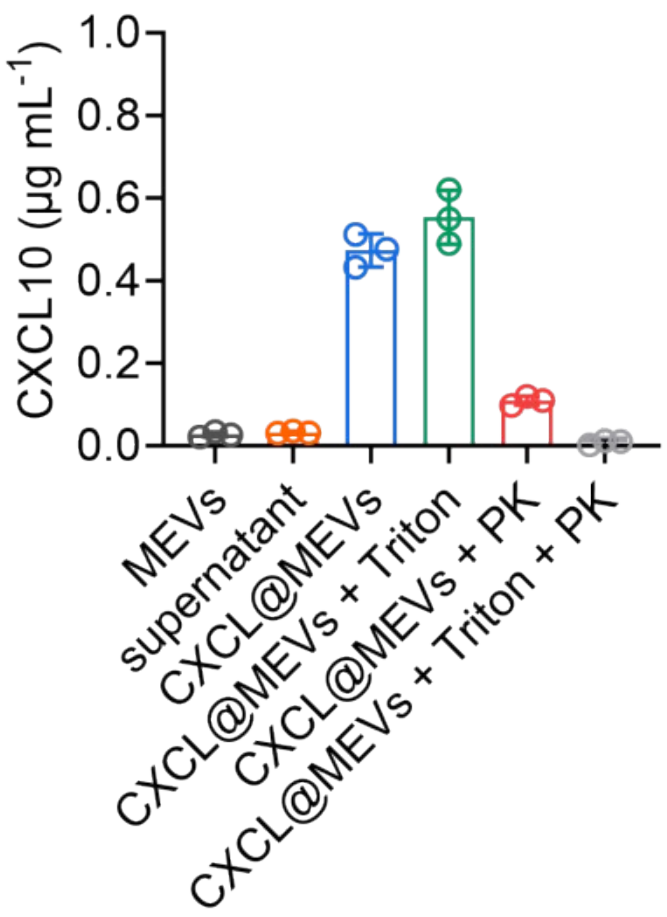

Fig. S2. CXCL10 levels in MEVs, supernatant, CXCL@MEVs, CXCL@MEVs treated with Triton X-100, CXCL@MEVs treated with proteinase K, and CXCL@MEVs treated with Triton X-100 plus proteinase K. Data are shown as mean ± SD, n = 3.

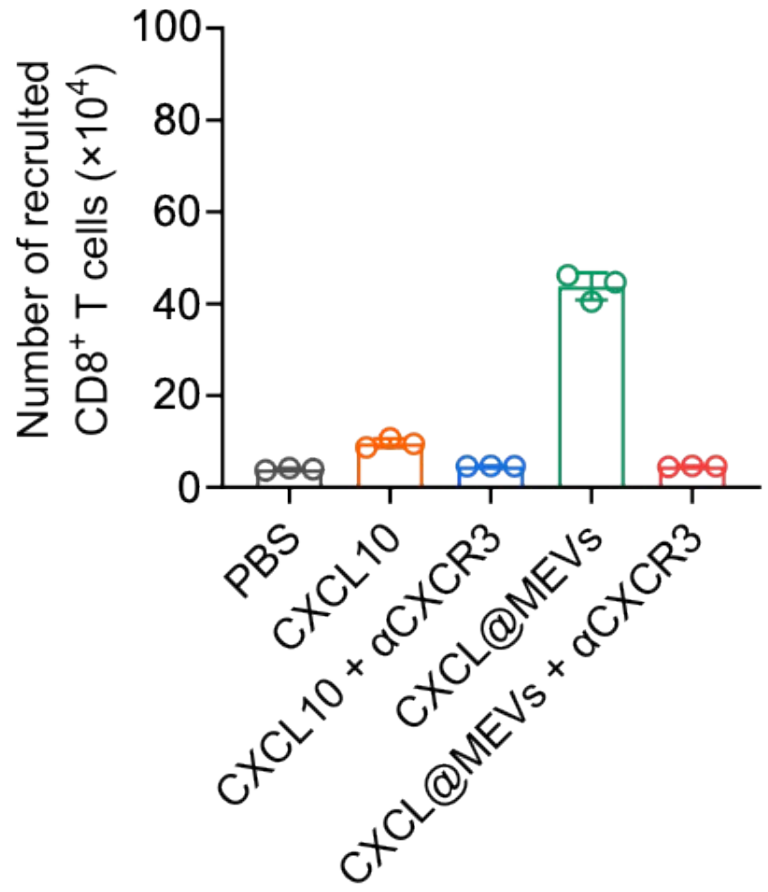

Fig. S3. CD8⁺ T cells were pretreated with an anti-CXCR3 blocking antibody before Transwell migration analysis. PBS, recombinant CXCL10, MEVs or CXCL@MEVs were added to the lower chambers as chemoattractants. Data are shown as mean ± SD, n = 3.

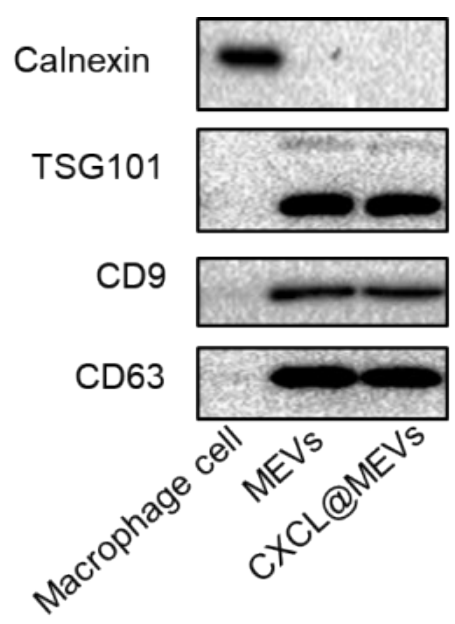

Fig. S4. Representative immunoblots of parental M1 macrophage lysates, MEVs and CXCL@MEVs for EV-enriched markers (CD9, CD63 and TSG101) and the negative marker calnexin.

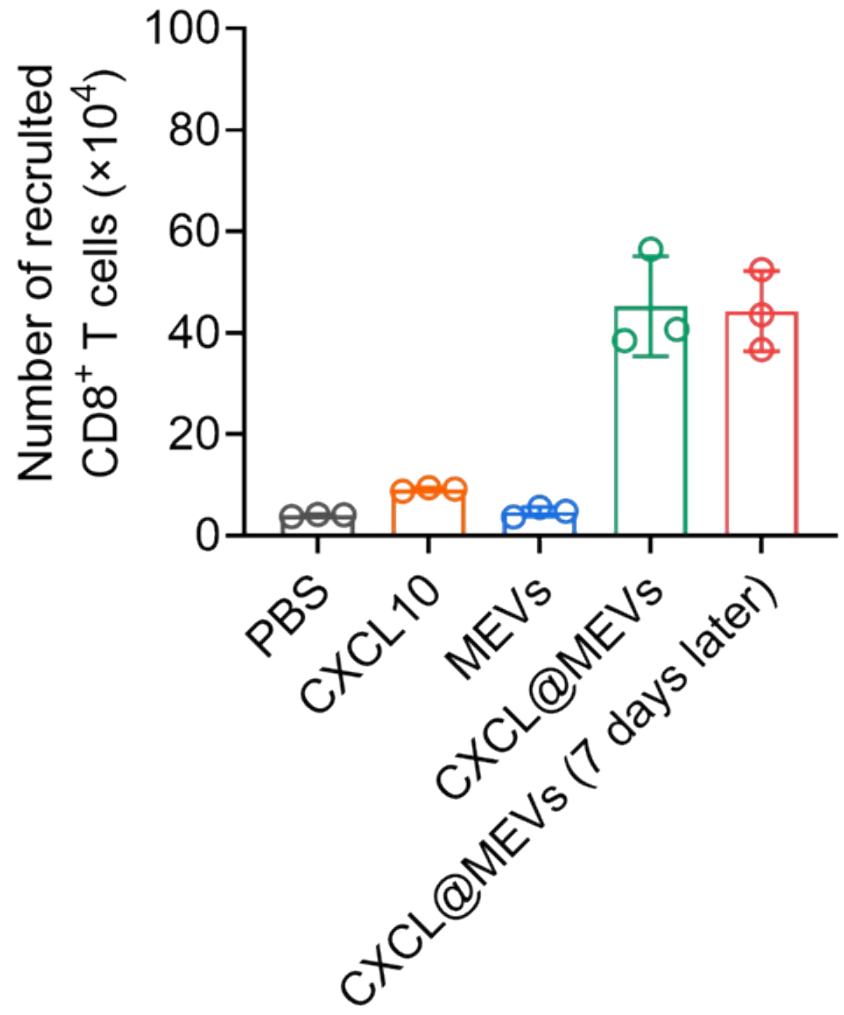

Fig. S5. CD8⁺ T-cell recruitment induced by PBS, free CXCL10, MEVs, freshly prepared CXCL@MEVs and CXCL@MEVs stored at 4 °C for 7 days. Data are shown as mean ± SD, n = 3.

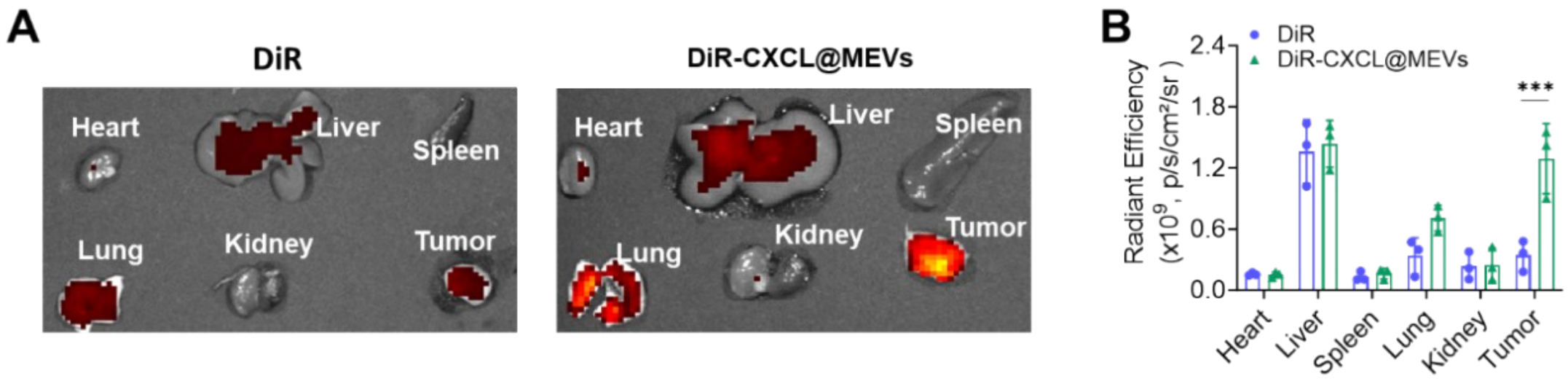

Fig. S6. In vivo biodistribution of DiR-labelled CXCL@MEVs after intravenous administration. (A) Representative ex vivo fluorescence images of heart, liver, spleen, lung, kidney and tumor collected 24 h after intravenous injection of free DiR or DiR-labelled CXCL@MEVs into PANC02 tumor-bearing mice. (B) Quantification of fluorescence intensity in tumors and major organs. Data are presented as mean ± SD; Statistical significance was calculated by one-way or two-way ANOVA with Tukey's post hoc test. *$P < 0.05$, **$P < 0.01$, ***$P < 0.001$.

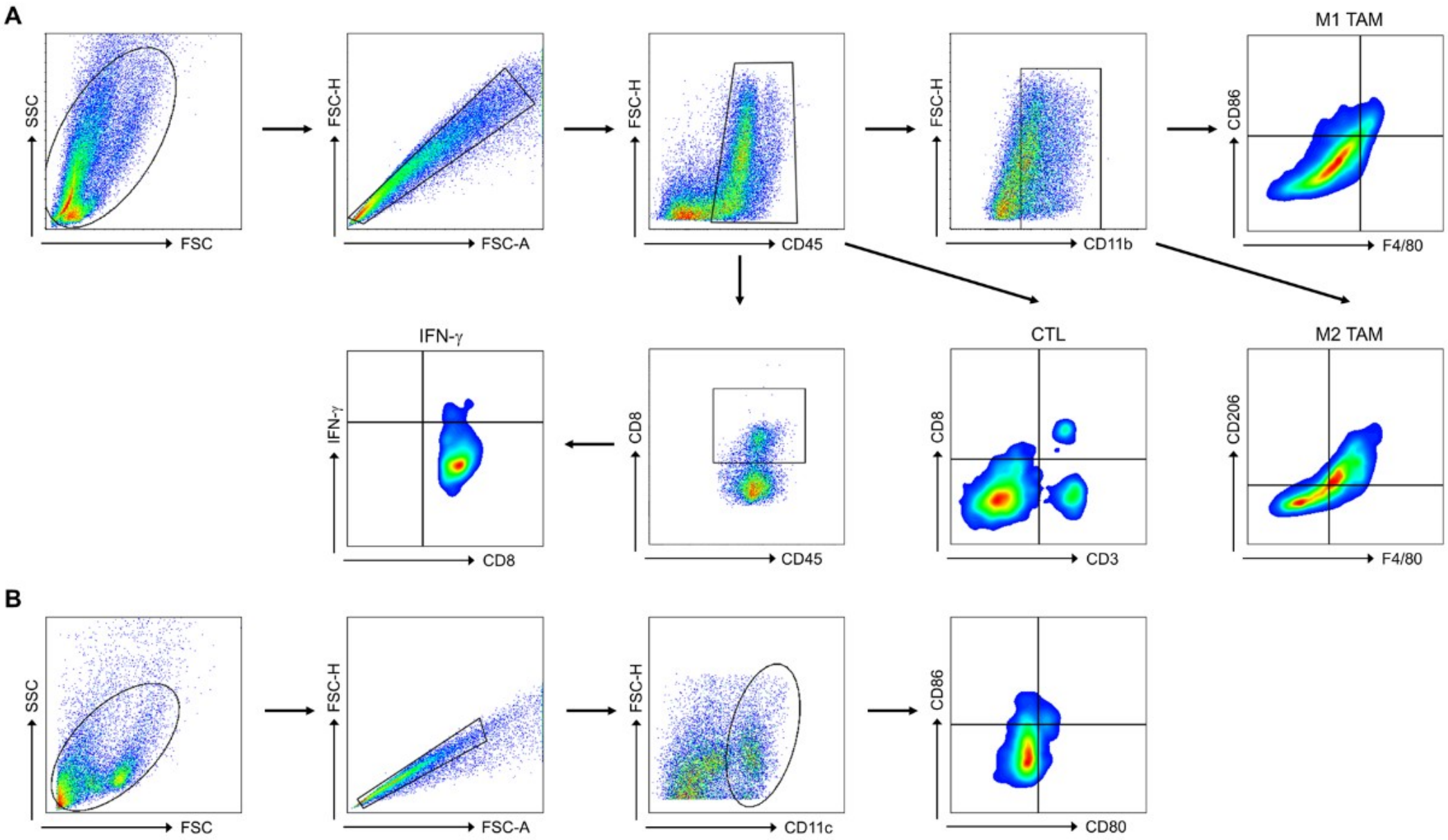

Fig. S7. Flow-cytometry gating strategies. (A) Representative gating strategy for tumor-infiltrating immune cells, including $CD45^+$ leukocytes, $CD45^+CD11b^+F4/80^+$ TAMs, M1-like TAMs, M2-like TAMs, $CD45^+CD3^+CD8^+$ CTLs and IFN-γ^+ $CD8^+$ T cells. (B) Representative gating strategy for dendritic-cell maturation based on CD11c, CD80 and CD86 expression.

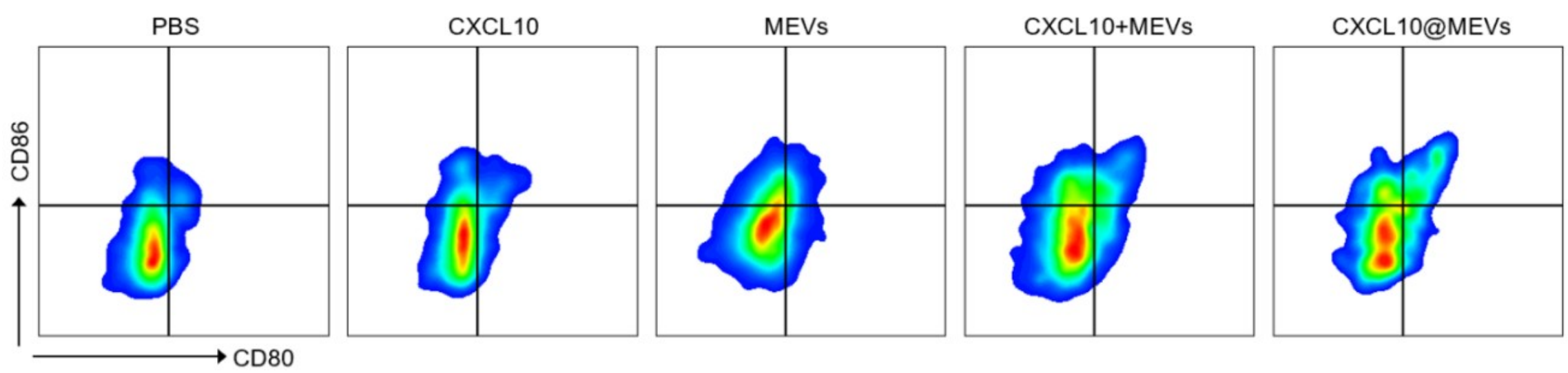

Fig. S8. Representative flow cytometry plots of mature DCs in the tumor-draining lymph nodes of mice bearing PANC02 subcutaneous tumors treated with the indicated formulations.

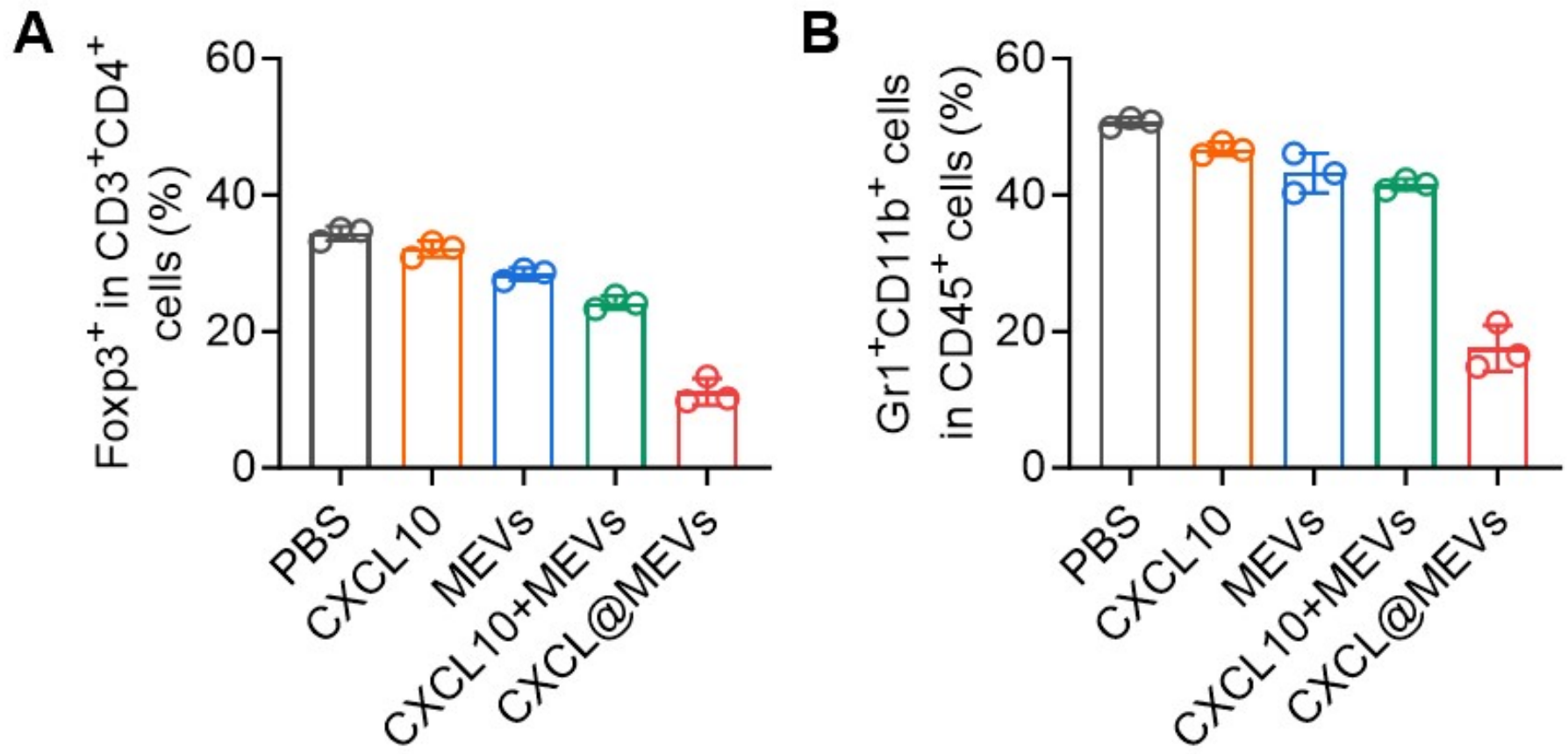

Fig. S9. Quantification of Tregs (A) and MDSCs (B) in the tumor sites at 72 h post-treatment with different formulations from mice bearing subcutaneous PANC02 tumors.

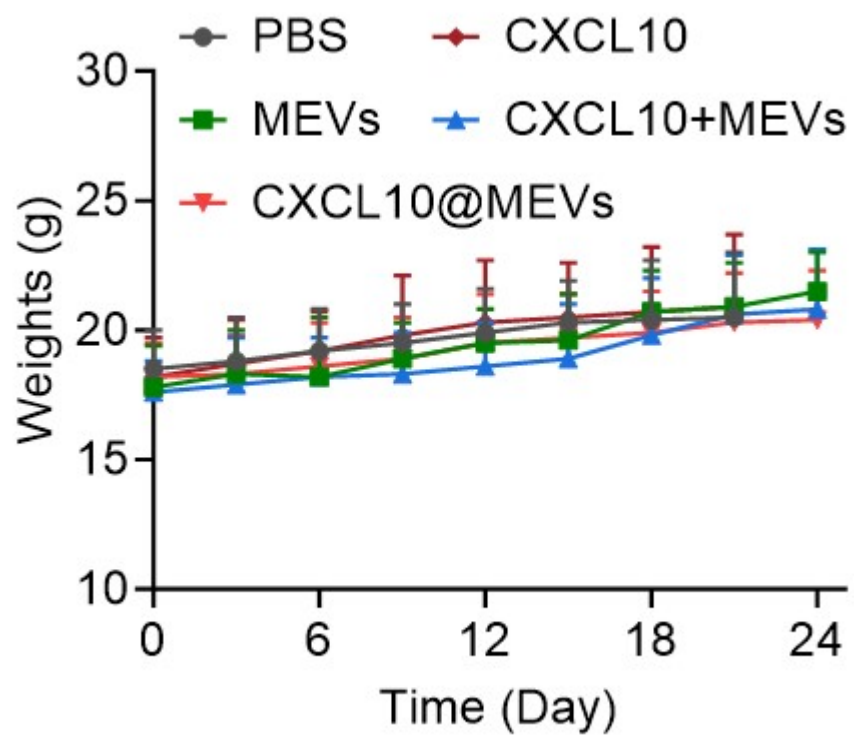

Fig. S10. Body weight of PANC02 subcutaneous tumor-bearing mice treated with the indicated formulations.

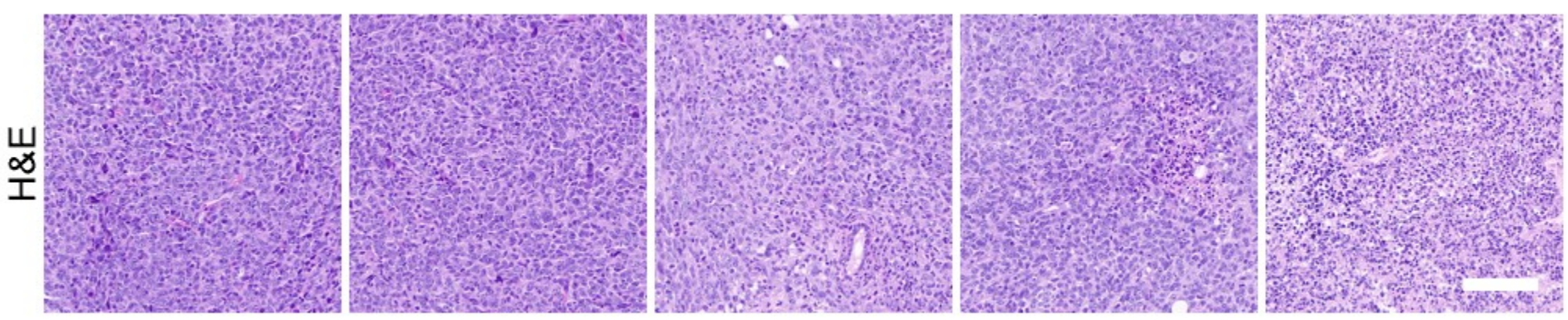

Fig. S11. H&E analysis of tumor tissues from mice bearing PANC02 subcutaneous tumors at day 24 post-injection of CXCL@MEVs. Scale bar, 200 µm.

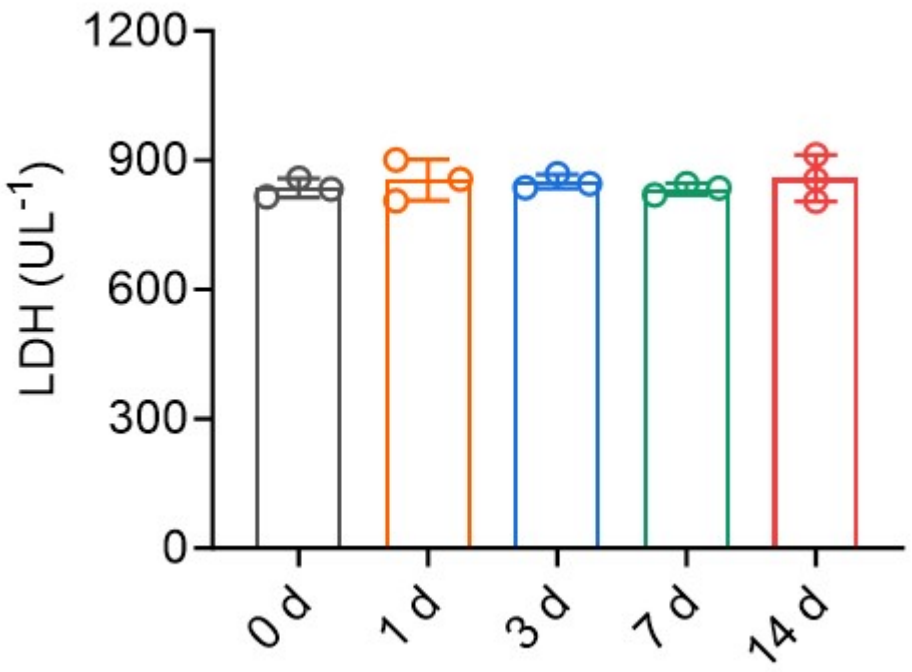

Fig. S12. LDH (lactate dehydrogenase) levels in mice treated with CXCL@MEVs.

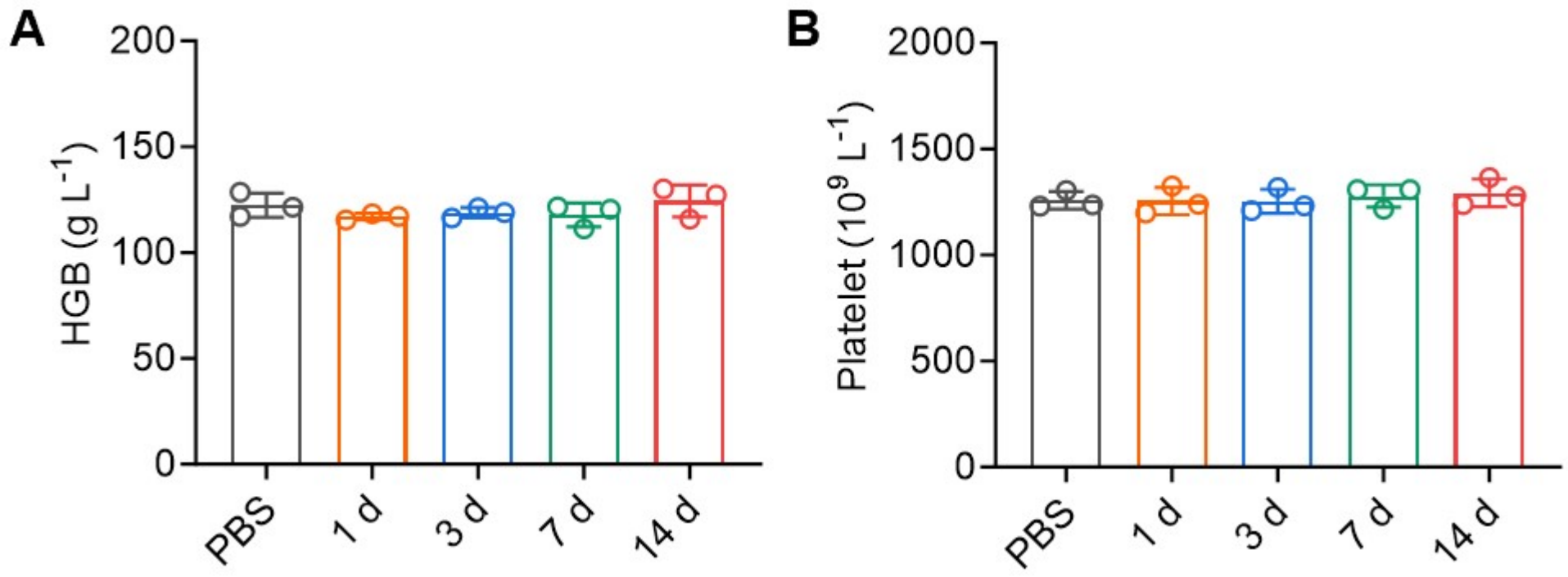

Fig. S13. Hemoglobin (HGB) levels (A) and platelet counts (B) in the blood of mice treated with CXCL@MEVs.

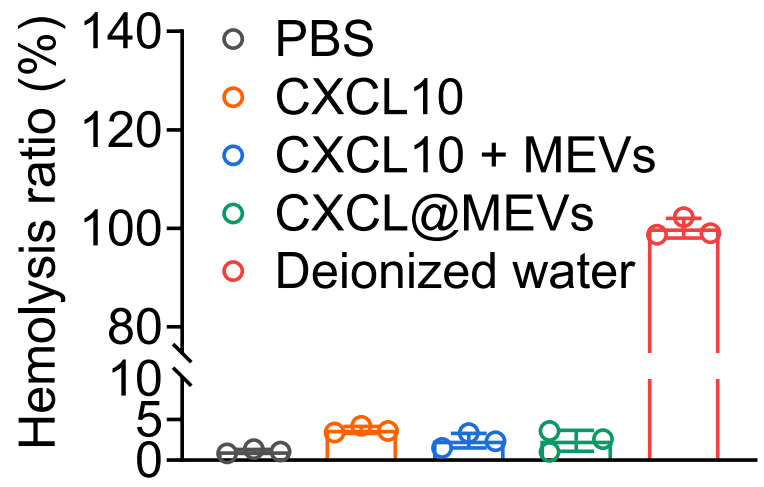

Fig. S14. Hemocompatibility of CXCL@MEVs. Hemolysis rates of erythrocytes after incubation with the indicated formulations. PBS and deionized water/0.1% Triton X-100 were used as the negative and positive controls, respectively. Data are presented as mean ± SD (n = 3). Statistical significance was calculated by one-way ANOVA with Tukey's post hoc test.

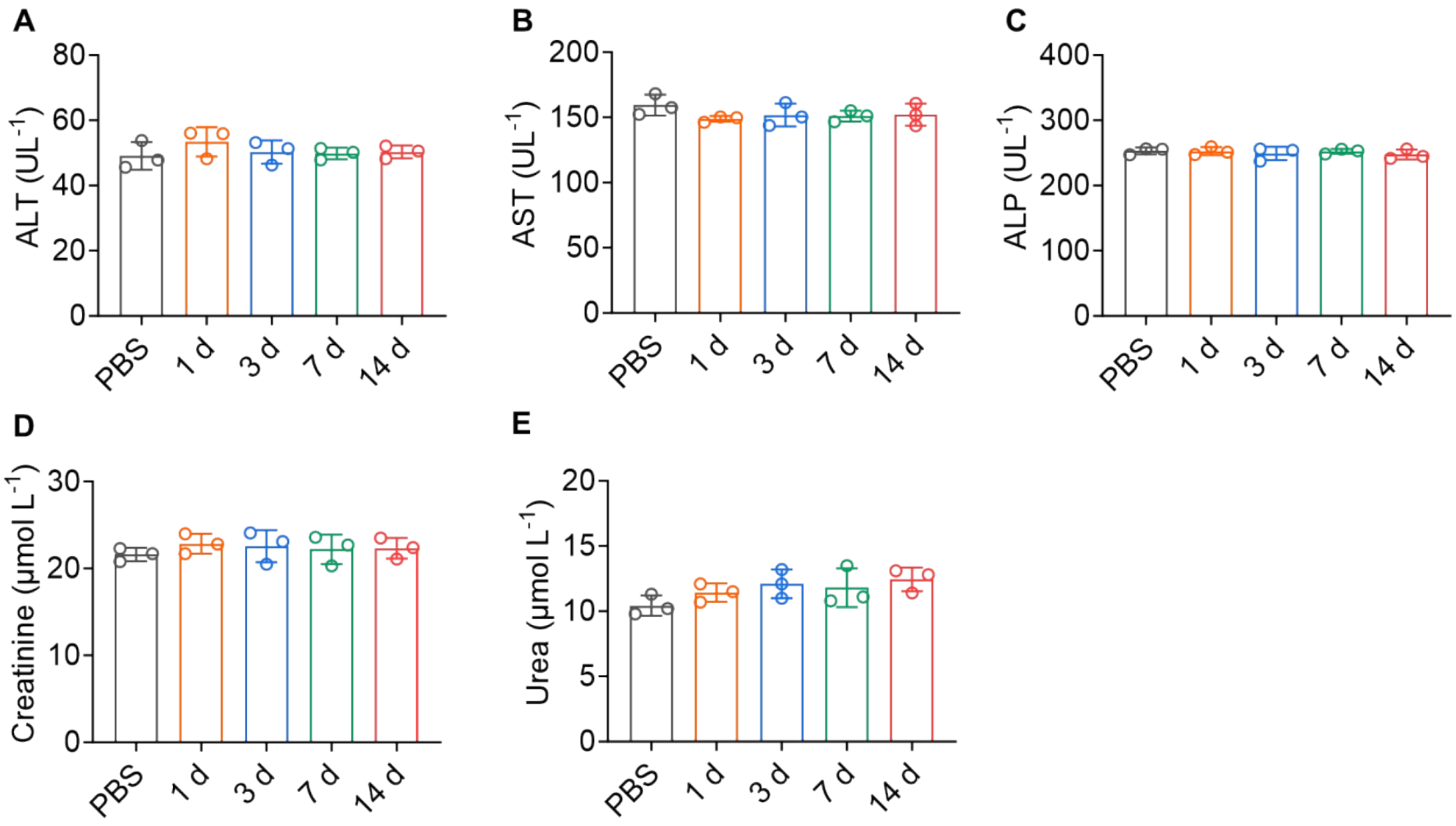

Fig. S15. Serum biochemical analysis following systemic administration of CXCL@MEVs. Serum levels of (A) alanine aminotransferase (ALT), (B) aspartate aminotransferase (AST), (C) alkaline phosphatase (ALP), (D) creatinine and (E) urea in mice after the indicated treatments. Data are presented as mean ± SD. Statistical significance was calculated by one-way ANOVA with Tukey's post hoc test.

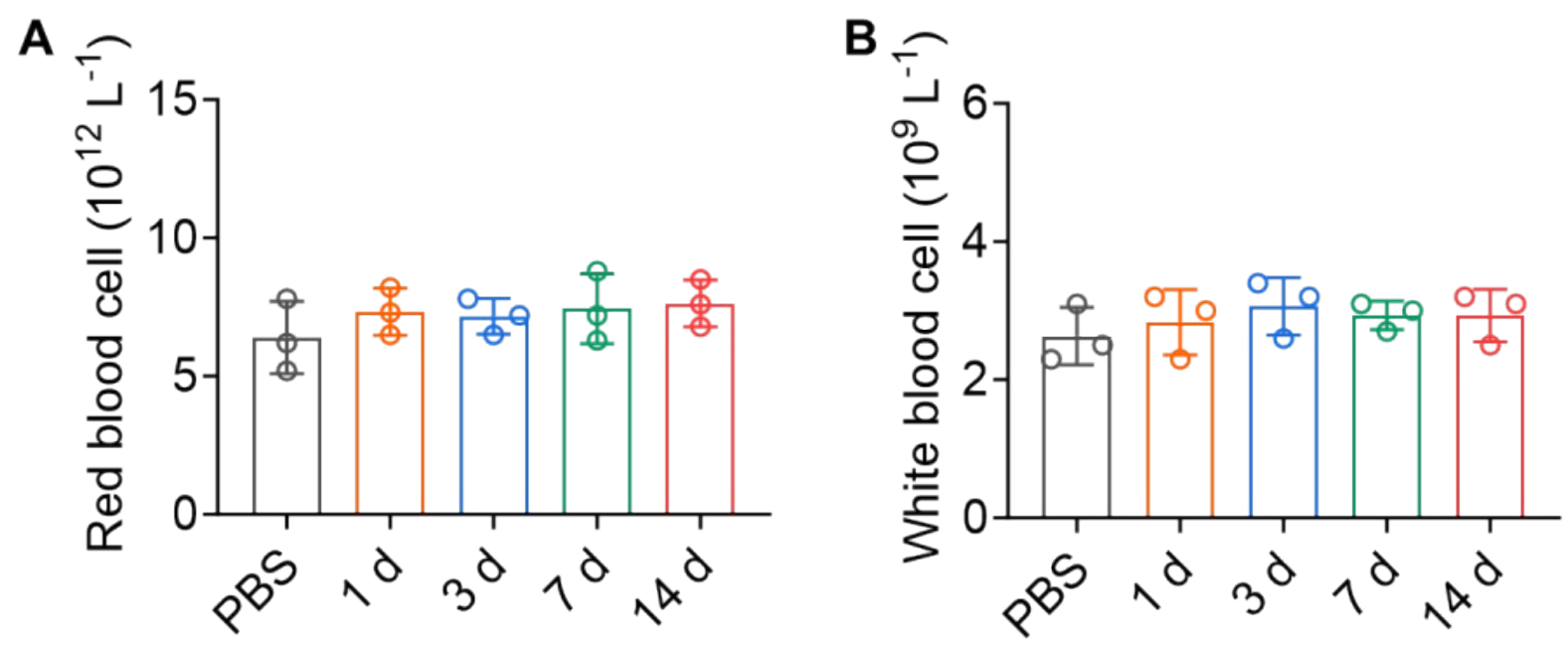

Fig. S16. Hematological analysis following systemic administration of CXCL@MEVs. (A) Red blood cell (RBC) and (B) white blood cell (WBC) counts in mice after the indicated treatments. Data are presented as mean ± SD. Statistical significance was calculated by one-way ANOVA with Tukey's post hoc test.

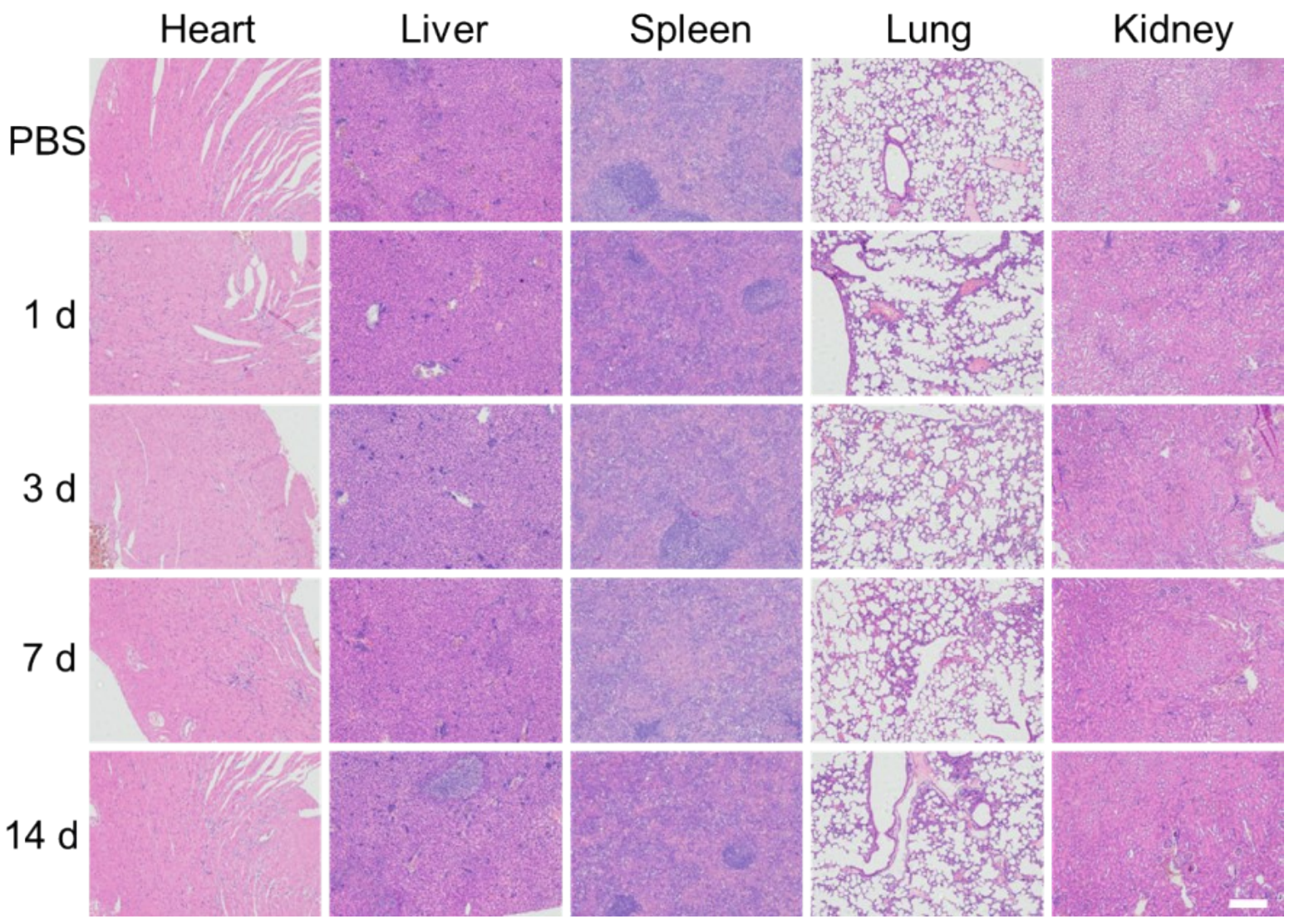

Fig. S17. Histological evaluation of major organs following CXCL@MEV treatment. Representative H&E-stained sections of the heart, liver, spleen, lung and kidney from PANC02 tumor-bearing mice after the indicated treatments. Scale bar, 200 μm**.**
